# Which School Community Agents Influence Adolescents’ Motivational Outcomes and Physical Activity? Are More Autonomy-Supportive Relationships Necessarily Better?

**DOI:** 10.3390/ijerph15091875

**Published:** 2018-08-30

**Authors:** Javier Sevil, Luis García-González, Ángel Abós, Eduardo Generelo Lanaspa, Alberto Aibar Solana

**Affiliations:** 1 Faculty of Health and Sport Sciences, Department of Didactics of the Musical, Plastic and Corporal Expression, University of Zaragoza, 22001 Huesca, Spain; lgarciag@unizar.es (L.G.-G.); aabosc@unizar.es (Á.A.); generelo@unizar.es (E.G.L.); 2 Faculty of Social Sciences and Humanities, Department of Didactics of the Musical, Plastic and Corporal Expression, University of Zaragoza, 22003 Huesca, Spain; aibar@unizar.es

**Keywords:** physical activity, health promotion, school, autonomy support, autonomous motivation, basic psychological needs, intention to be physically active, adolescence, self-determination theory

## Abstract

The first aim of this work was to examine the independent influence of students’ perceived autonomy support for leisure-time physical activity (LTPA), from different school community agents, on motivational outcomes in a LTPA context and objective PA levels. Using both a variable- and person-centered approach, the second aim was to examine how different combinations of autonomy-support were associated with students’ motivational outcomes in a LTPA context and PA levels. A sample of 178 secondary students (*M* = 13.26 ± 0.64) participated in the study. Autonomy support for LTPA from the PE teacher, mother, father, and peers were the only agents that significantly and positively predicted motivational outcomes in a LTPA context and PA levels. While the two- and three-way interactions of some of these four significant sources significantly increased the explained variance of some motivational outcomes, the plots revealed that the lowest values of motivational outcomes were associated with low values of perceived autonomy support. A cluster analysis revealed five profiles. The “high autonomy support” group reported the most optimal outcomes, whereas the “low autonomy support” group showed the opposite pattern. However, mixed autonomy support profiles did not differ in any of the outcomes. The adoption of a whole-of-school approach seems decisive to empower adolescents to be active in and out of school.

## 1. Introduction

Despite the well-known health benefits of regular physical activity (PA) [1], a significant proportion of adolescents do not meet PA recommendations [2]. Particularly, the transition from childhood to adolescence has been characterized as a critical period when PA levels progressively decrease [3], which could be explained by biological, psychological, and social changes [4]. For example, and considering social differences, support for PA from parents seems to decrease in the transition to adolescence, whereas the influence of friends on PA become increasingly more important [5]. Based on previous reviews [6,7], not only socio-cultural determinants (i.e., causally related factors) but also individual determinants (i.e., associated factors) have been identified as potential correlates of PA behavior among adolescents. 

Self-Determination Theory (SDT) [8] is a widely used framework that has been used to understand the social and psychological factors that influence PA behavior. SDT posits that autonomy (i.e., sense of choice and volition in one’s actions), competence (i.e., sense of efficacy and confidence in achieving desired outcomes), and relatedness (i.e., sense of belongingness and connectedness to significant others) are three basic psychological needs (BPNs) that must be fulfilled to achieve optimal psychological development and well-being [9]. The degree to which BPNs are satisfied determine, in turn, the type of motivation, which varies along a continuum from more to less internalization (i.e., autonomous motivation, controlled motivation, and amotivation) [9]. Autonomous motivation (i.e., undertaking an activity due to internal reasons such as interest, enjoyment, core goals, personal values, and benefits), represents the most self-determined form of motivation, and it is comprised of intrinsic motivation, integrated regulation, and identified regulation. Students’ satisfaction of BPNs in leisure-time PA (LTPA) has been positively associated with autonomous motivation for LTPA [10,11], which, in turn, has been positively associated with positive behavioral outcomes such as intention to be physically active [12] and PA levels [13]. 

According to SDT, social-contextual factors such as autonomy-support for LTPA (i.e., providing adolescents with choices, options, and opportunities to do PA) from significant others may have an influence on students’ motivational outcomes and, consequently, may enhance initiation and long-term maintenance of PA [14,15]. Although most studies have demonstrated that students’ perceived autonomy support for LTPA from some significant others, such as parents, physical education (PE) teacher, or peers, was independently and positively associated with BPN satisfaction in LTPA [10], autonomous motivation for LTPA [16], intention to be physically active [17], self-reported PA [18], and objective PA levels [19], only three studies to date have simultaneously analyzed these three sources of autonomy support for LTPA [18,19,20]. A few studies in the context of LTPA showed no relationship between autonomy support from the PE teacher and BPN satisfaction [20], autonomous motivation [20], intention to be physically active [20], and subjective PA levels [10,20,21]. In addition, one study also reported that autonomy support for LTPA from parents and peers was not a significant predictor of self-report and objective PA levels [22]. Given that different mixed results have been reported and most previous studies have used self-reported PA, further studies using objective measures of PA are required to gain insight into the relationship between all these variables. Despite mothers and fathers playing different roles in their children’s PA levels [23], only one study to date has examined autonomy support for LTPA including mothers and fathers separately, showing a weak association with autonomous motivation for LTPA and self-reported PA levels [16]. Moreover, little is known about the influence of autonomy support for LTPA from other significant sources of the school community such as from teachers of different disciplines and tutors, who play a key role in adolescents’ learning process. Identifying the significant sources of autonomy support for LTPA in the school community can provide insights into the choice of the social agents who should be mainly involved in the design of school-based PA interventions.

There is another important question about the combined effects of different sources of autonomy support that remains unanswered in literature. It is unclear whether the additional number of autonomy-supportive relationships displays more beneficial effects on students’ motivational outcomes in a LTPA context and PA levels. This question has been addressed in other contexts by testing the interactive effects of different influential sources of support e.g., [24] and/or using a person-centered approach e.g., [25]. Additive and threshold models are used to provide explanations to clarify this issue. According to the additive model, each additional source of support matters, while the threshold model posits that each additional source of support is redundant and does not contribute to an incremental effect on selected outcomes [26]. 

Despite a large body of research having examined the independent influence of social agents on adolescents’ motivational outcomes, a limited number of studies have examined the interaction effects of multiple social agents. For example, two studies among youth soccer players found that perceiving a combination of at least two of the three types of social relationships from peers (i.e., peer acceptance and friendship quality) and parents (i.e., mother-child or father-child relationship quality) were positively associated with higher perceived values of competence, self-determined motivation (only in the mother-child relationship model), enjoyment [27], and continued sport participation [28], mainly supporting the threshold model. In these studies, two- and three-way interactions between different types of social relationships significantly added an extra range of between ∆*R*^2^ = 0.03 and ∆*R*^2^ = 0.06 to the prediction of motivational outcomes. In the same way, a previous study, which examined the combined effects of youth athletes’ perceived autonomy support from different social agents (e.g., father, mother, and coach) on motivational outcomes, explained small additional amounts of variance rather than considering exclusively independent effects (total ∆*R*^2^ = 0.03) [24]. The interaction plots showed that either of the two-social agent combination possibilities (i.e., father and mother, mother and coach, and coach and father) provided similar predicted values in self-determined motivation to the combination of the three sources of autonomy support, which also seems to support the threshold model. It is also noteworthy that the lowest predicted values of motivational outcomes, indicated in the latest studies [24,27,28], occurred when athletes perceived low support from all sources. To our knowledge, these studies have mainly focused on youth sport participants who already participated in PA out of school. Given that schools represent the only setting that provides an opportunity to reach all adolescents, further research to examine the combined effect of the most significant social agents of the school community on adolescents’ motivation in a LTPA context and PA levels is fully warranted.

Individuals can simultaneously perceive several sources of support [25], that is why a person-centered approach has also been applied in this field to further understand the dichotomy between the threshold and the additive model. This approach should allow us not only to identify different combinations of autonomy-supportive relationships, but also to examine whether these resulting profiles differ in terms of motivational outcomes in a LTPA context and PA levels. Two previous person-centered studies that addressed this question in educational settings found that students, who perceived all sources of support or relatedness support, reported better results in perceived scholastic competence, behavioral conduct, adjustment problems [26], and emotional and behavioral engagement [29], providing evidence to support the additive model. It is important to note that in most of the study variables of Laursen and Mooney [26], no significant differences were found between the profiles with zero, one, or two high positive relationships, suggesting that only one more additional source of support is not necessarily better.

Consistent with Laursen and Mooney [26], two more recent studies using cluster analysis among secondary [25] and university students [30] showed that more sources of autonomy support in the educational context are not always better. In both studies [25,30], the groups that perceived moderate to high autonomy support from all significant sources reported better results in selected outcomes than the groups who perceived low autonomy support from those sources (additive model). However, mixed results were found in both studies between the profiles with two and three autonomy-supportive relationships, increasing the controversy between the additive and threshold models. In the study of Guay et al. [25], autonomy support by teachers and mothers was sufficient to sustain autonomous motivation and competence (threshold model), but was not enough to obtain higher academic achievement, for which autonomy support from all sources (adding the father) was required (additive model). In the study of Ratelle et al. [30], autonomy support from a romantic partner buffered the lack of autonomy support from the other sources (parents and friends) in terms of academic satisfaction (threshold model), whereas all sources of autonomy support (i.e., parents, friends, and romantic partner) were required to foster satisfaction with life and negative/positive affect (additive model). Moreover, according to Ratelle et al. [30], the group of students who perceived all sources as highly autonomy-supportive showed significantly better results in terms of satisfaction with life and negative/positive affect than the group of students who perceived all sources as moderately autonomy-supportive. Therefore, not only the number of autonomy-supportive relationships (i.e., three supports) and the type of agent that supports autonomy (i.e., parents, friends, and romantic partner) seems to be important, but also the quantity (i.e., moderate or high) of that autonomy support from each agent. In addition, mixed results were found in these two studies that compared the profiles with low autonomy support from all respective sources and the profiles with at least one salient source of autonomy support. In the study of Guay et al. [25], the group with moderate autonomy support from teacher and mother reported higher autonomous motivation, competence, and achievement than the group that perceived all sources as non-autonomy-supportive (additive model). On the contrary, in the study of Ratelle et al. [30], no differences were found between the group with low autonomy support from all sources and the group with one or two salient sources of autonomy support (threshold model).

The more recent cluster study conducted by Gardner, Magee and Vella [31] on youth sports revealed that although the group with higher levels of four social relationships (parental support, coach-athlete relationship quality, friendship quality, and peer acceptance), reported better results in adolescents’ enjoyment and continued sport participation than the group with the lowest levels of support from all agents (additive model), no differences were found in the study variables between the first mentioned group and the profile characterized only by higher levels of coach-athlete relationship quality (threshold model). No differences were found in adolescents’ enjoyment, either, between the profile that received the lowest levels of support and the profile characterized by high friendship quality support. All these results suggest the importance of considering not only the number of different social relationships but also with whom the interaction occurs. As seen in literature, recent studies seem not to clarify the controversy regarding the effect on individuals’ outcomes of adding more autonomy-supportive relationships. Therefore, further research is warranted to better understand the potential co-occurrence of relationships between different social agents and their influence on PA-related outcomes among adolescents.

### The Present Study

Grounded in SDT, the first aim of this study was to examine the independent influence of students’ perceived autonomy support for LTPA from different sources of the school community (i.e., PE teacher, teachers, mother, father, peers, and tutor) on BPN satisfaction in LTPA, autonomous motivation for LTPA, intention to be physically active, and PA levels. Based on most previous research e.g., [19], one would expect to find a significant positive relationship between autonomy support for LTPA from the PE teacher, mother, father, and peers, and motivational outcomes in a LTPA context and PA levels, respectively. In view of other studies that have evaluated different types of teacher support for PA (e.g., logistic support, modeling, involvement support, etc.) e.g., [32], and considering the amount of time that teachers spend with their students and, in particular, the key role played by the tutor in students’ personal lives and educational process, it was hypothesized that the teachers and tutor would also be positively associated with selected outcomes, although to a lesser extent than the other social influences (Hypothesis 1). The second aim was to further explore the combination of autonomy-supportive relationships that significantly predicted adolescents’ PA motivation and PA levels by means of a (1) variable- and a (2) person-centered approach. More specifically, (1) we investigated the interaction effects of the different sources of autonomy support for LTPA on selected outcomes, and (2) we identified distinct profiles of autonomy-supportive relationships and examined their differences in terms of adolescents’ motivational outcomes in a LTPA context and PA levels. These two different but complementary types of approaches were conducted to answer the question about whether the number of autonomy-supportive relationships matters in adolescents’ motivational experiences in a LTPA context and PA levels. One would expect the interaction of autonomy support from some of these social agents to have significantly added explained variance to the prediction of PA-related behaviors, above and beyond their independent effects. Given the exploratory nature of this study, we did not formulate hypotheses regarding the possible combination of social agents that may interact to predict higher values in dependent variables. Nevertheless, if a significant two-, three-, or four-way interaction emerged, the predicted values of motivational outcomes in a LTPA context and PA levels would be expected to be higher when adolescents have perceived most of these sources of autonomy support. On the contrary, the lowest predicted values would be expected to be found in adolescents who have received low autonomy support from these sources (Hypothesis 2a). As far as profiles are concerned, and consistent with previous studies [25,30,31], one would expect to find at least three types of profiles: a “high autonomy support” profile characterized by students who perceived moderate or high autonomy support from all social agents, a “low autonomy support” profile that showed an opposite pattern, and one or several “mixed autonomy support” profile(s) characterized by students who have perceived a moderate or high autonomy support from one or several sources. Although one would expect students, who reported high autonomy support from all significant sources, to display the most adaptive pattern of outcomes in comparison to students who reported low autonomy support from all of them, it is not clear from previous studies whether autonomy support from the most relevant sources of autonomy support would be sufficient to sustain students’ motivational outcomes and PA levels. Consequently, no hypothesis was formulated regarding the additive or the threshold effect that other “mixed autonomy support” profiles might have (Hypothesis 2b). 

## 2. Method

### 2.1. Design, Participants and Procedure 

A cross-sectional design was used to investigate all research questions. A total of 225 8th grade students from two secondary schools in Huesca (Spain) were initially invited to participate in this study (*M* = 13.06 ± 0.61 years). Participation was entirely voluntary and confidential. Written informed consent from both parents and adolescents was obtained from 210 students (93.33% response rate, 47.1% boys). Firstly, the students wore an accelerometer, for a 7-day period, to measure PA levels. After that, a paper-and-pencil survey was administered to students in the classroom during a period of approximately 40 min (100% response rate) without the presence of teachers. A total of 26 students did not meet the accelerometer inclusion criteria and were excluded (*n* = 184; 87.6% valid rate). After removing univariate and multivariate outliers, the final sample was composed of 178 secondary school students (38.3% boys, *M* = 13.26 ± 0.64 years), most of whom were Caucasian (83.2%). The Ethics Committee for Clinical Research of Aragon (CEICA) approved all procedures of this study. 

### 2.2. Instruments

#### 2.2.1. Perceived Autonomy Support for LTPA 

Students’ perceptions of autonomy support for LTPA from different sources of the school community (i.e., PE teacher, teachers, mother, father, peers, and tutor) were measured by the Spanish version [33] of the Perceived Autonomy Support Scale for Exercise Settings (PASSES) [18,34]. The 12 item-scale was answered separately for each social agent (e.g., “My PE teacher/teachers/mother/father/tutor/peers encourage(s) me to do active sports in my free time”). Students’ responses were rated on a 7-point Likert scale ranging from 1 (strongly disagree) to 7 (strongly agree). In this study, Cronbach’s alpha values ranged from 0.91 to 0.96 across agents. 

#### 2.2.2. Basic Psychological Need Satisfaction in LTPA 

Students’ perceptions of autonomy, competence, and relatedness satisfaction in LTPA were assessed using the Spanish version [35] of the Basic Psychological Needs in Exercise Scale (BPNES) [36]. The scale consists of 12 items (four items per factor) that assess: autonomy (e.g., “I feel very strongly that I have the opportunity to make choices with respect to the way I exercise”), competence (e.g., “I feel that I execute the exercises of my training program very effectively”), and relatedness (e.g., “I feel that I mix with the other exercise participants in a very friendly way”), introduced by the statement “When I do physical activity…”. The scale was rated on a 5-point Likert-type scale ranging from 1 (strongly disagree) to 5 (strongly agree). Considering previous studies on LTPA e.g., [11], scores for autonomy, competence, and relatedness satisfaction were combined into a single composite score of BPN satisfaction. In this study, Cronbach’s alpha value was 0.89 for BPN satisfaction.

#### 2.2.3. Autonomous Motivation for LTPA 

Students’ perceptions of autonomous motivation in LTPA were assessed using the Spanish version [37] of the Behavioral Regulation in Exercise Questionnaire [38]. From the 23 items of this scale, in this study we only assessed the 11 items (three factors) that made up autonomous motivation: intrinsic motivation (four items; e.g., “I get pleasure and satisfaction from participating in PA”), integrated regulation (four items; e.g., “I consider PA to be a fundamental part of who I am”), and identified regulation (three items; e.g., “I value the benefits of PA”). Following the statement: “Why do you engage in PA?” students were asked to rate each item on a 4-point Likert-type scale ranging from 1 (not true for me) to 4 (very true for me). Considering previous studies on LTPA e.g., [39], and according to the tenets of SDT, average values of intrinsic motivation, integrated regulation, and identified regulation were used to calculate a composite variable of autonomous motivation. In this study, Cronbach’s alpha value was 0.86 for autonomous motivation. 

#### 2.2.4. Intention to Be Physically Active. 

Students’ perceptions of intention to be physically active were assessed using the Spanish version [40] of a three item-scale developed by Hagger et al. [18]. The scale is rated on a 7-point scale ranging from 1 (strongly agree) to 7 (strongly disagree) end points. In this study, Cronbach’s alpha value was 0.94 for intention to be physically active.

#### 2.2.5. MVPA Levels 

The average daily moderate-to-vigorous PA (MVPA) was objectively assessed using Actigraph GT3X accelerometers (Actigraph, Pensacola, FL, USA) for a 7-day period. The epoch length was set at 15 s as recommended for adolescents [41]. Daily MVPA was estimated using Evenson cut-points [42]. The inclusion criteria for this study were to wear the accelerometer at least three weekdays for a minimum of 10 h and one weekend-day for a minimum of 8 h [43,44]. Students wore accelerometers on the right side of the hip (anterior to the iliac crest) during waking hours (except for water-based activities, bathing, and sleeping).

#### 2.2.6. Demographic Variables 

Students’ age, gender, and socio-economic status (SES) were also self-reported. A composite SES index ranging from 0 to 9 was calculated adding the items of the Family-Affluence Scale II (FAS II) [45]. 

### 2.3. Data Analysis

Firstly, Kolmogorov-Smirnov tests were used to investigate if data were normally distributed (*p* > 0.05). Secondly, descriptive statistics (mean and standard deviation), internal consistency (via Cronbach’s alpha coefficient), and correlational analyses (via Pearson correlation coefficient) were performed for all variables of the study. In keeping with the first aim of the study, univariate regression analyses were run to examine the independent association between students’ perception of autonomy support from the PE teacher, teachers, mother, father, peers, and tutor with a set of dependent variables (i.e., BPN satisfaction in LTPA, autonomous motivation for LTPA, intention to be physically active, and PA levels). After that, using only significant univariate correlates (i.e., those that significantly predicted dependent variables), a series of multivariate hierarchical regression analyses [46] were performed to examine the independent and interactive influence of different sources of autonomy support on motivational outcomes and PA levels. Thus, all previous significant sources of autonomy support were entered at step one of all hierarchical regression analyses to examine their independent influence. To analyze whether combining different sources of autonomy support increased the explained variance of the dependent variables beyond the independent effects, a different set of interactions were calculated in subsequent steps according to the second aim of the study (Hypothesis 2a). It is important to note again that only statistically significant sources of autonomy support, that showed an independent influence on most dependent variables at step one, were considered in the subsequent steps. Following several authors, sources of autonomy support were centered, and the interaction terms were created using the product of centered-variables [46,47]. Change in R^2^ was examined to determine the explained variance added by the interaction terms at each step [48]. Significant interactions were plotted using high (+1 SD) and low (−1 SD) values of each source of autonomy support to facilitate interpretation [46]. To further examine the specific relationship of the two-way interaction effect, simple slope tests were conducted [47,49]. A slope difference test was also carried out to analyze significant differences between each pair of regression slopes in the three-way interactions [47,50].

Based on the sources of autonomy support for LTPA that independently influenced dependent variables, a cluster analysis was also conducted in the second aim to identify profiles of students’ autonomy support for LTPA. Each source of autonomy support for LTPA was standardized, and univariate and multivariate outliers were identified and removed to perform cluster analysis [51]. Consistent with Garson [52], a two-step procedure, combining hierarchical and non-hierarchical clustering methods, was used to define the number of clusters. Using Ward’s linkage method, a hierarchical cluster analysis was conducted to compute initial cluster centers in a first-step procedure. Three- to six- cluster solutions were inspected to identify the percentage of explained variance, which should be at least 50% for each source of autonomy support to be considered for further analysis [53]. A non-hierarchical k-means cluster analysis was carried out using cluster centers of each possible number of profiles in a two-step procedure [54]. A double-split cross-validation procedure was followed to examine the reliability and stability of the final solution. The sample was randomly split into two halves (50%) and the full two-step procedure (i.e., Ward, followed by k-means) was applied again to each subsample. These two new cluster solutions were averaged to evaluate the degree of agreement in relation to the original cluster solution using Cohen’s kappa (K), which should report a value of at least 0.60 to be considered acceptable [54].

Finally, Chi-square tests between cluster solution and gender, and SES were conducted. In addition, to examine whether cluster profiles differed in terms of motivational outcomes in a LTPA context and PA levels, a multivariate analysis of variance (MANOVA) with Bonferroni-corrected post-hoc pairwise comparisons was conducted (Hypothesis 2b). Partial eta-squared (η^2^_p_) values were used to calculate effect size of these pairwise comparisons as follows: 0.01 = small effect, 0.06 = medium effect and 0.14 = large effect [55]. All analyses were conducted using SPSS version 21 (SPSS Inc., Chicago, IL, USA). 

## 3. Results

### 3.1. Research Question 1

The Kolmogorov-Smirnov tests confirmed the normal distribution of the data. Descriptive statistics and correlations between the study variables are reported in Table 1. Students’ perception of autonomy support for LTPA from all six sources were significantly and positively correlated with motivational outcomes and PA levels, with the exception of autonomy support for LTPA from the tutor, which was only significantly related to intention to be physically active. Autonomy support for LTPA from all six social agents showed low to moderate correlations, which revealed that students differentiate the amount of support from different agents, barring several exceptions where no relationships were found.

Univariate regression analysis showed that students’ perception of autonomy support for LTPA from the PE teacher, mother, father, peers, and teachers significantly and positively predicted BPN satisfaction and autonomous motivation for LTPA, intention to be physically active, and PA levels. After that, the hierarchical regression analysis showed that only autonomy support for LTPA from the PE teacher, mother, father, and peers significantly and positively predicted most of these dependent variables (see Table 2). Based on this, autonomy support for LTPA from teachers was not included in further analyses (i.e., subsequent interaction steps and cluster analysis). Specifically, in step 1, autonomy support for LTPA from the PE teacher significantly and positively predicted BPN satisfaction in LTPA (*R*^2^ = 0.18), autonomous motivation for LTPA (*R*^2^ = 0.02), intention to be physically active (*R*^2^ = 0.02), and PA levels (*R*^2^ = 0.03). Autonomy support for LTPA from the father significantly and positively predicted BPN satisfaction in LTPA (*R*^2^ =0.05), autonomous motivation for LTPA (*R*^2^ = 0.05), intention to be physically active (*R*^2^ = 0.05), and PA levels (*R*^2^ = 0.08). Autonomy support for LTPA from the mother significantly and positively predicted BPN satisfaction in LTPA (*R*^2^ = 0.02), autonomous motivation for LTPA (*R*^2^ = 0.14), and intention to be physically active (*R*^2^ = 0.12). Finally, autonomy support for LTPA from peers significantly and positively predicted BPN satisfaction in LTPA (*R*^2^ = 0.09), autonomous motivation for LTPA (*R*^2^ = 0.08), and in particular intention to be physically active (*R*^2^ = 0.16) and PA levels (*R*^2^ = 0.14) (see the standardized regression coefficients in Table 2). 

### 3.2. Research Question 2

#### 3.2.1. Hypothesis 2a: Variable-Centered Approach

**Figure 1:**
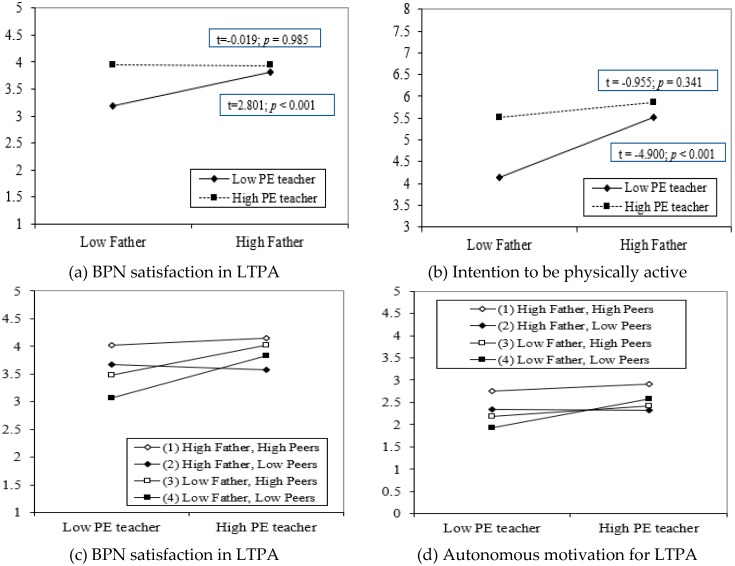
Note 1: Moderation effect between autonomy support from the PE teacher and father in predicting: (**a**) BPN satisfaction in LTPA and (**b**) intention to be physically active (two-way interaction with continuous moderator). Note 2: Moderation effect between autonomy support from the PE teacher, father, and peers in predicting: (**c**) BPN satisfaction in LTPA and (**d**) autonomous motivation for LTPA (three-way interaction with continuous moderators).

The inclusion of two-way and three-way interaction terms, in step 2 and step 3 respectively, significantly increased the amount of explained variance in all dependent variables (particularly from step 1 to step 2 in motivational outcomes in LTPA) (see Table 2). The two-way interaction plots and their simple slope tests showed that, when perceived autonomy support from the PE teacher and the father were low, the values of BPN satisfaction in LTPA and intention to be physically active were the lowest (BPN satisfaction; t = 2.801, *p* < 0.01; intention to be physically active: t = 4.900, *p* < 0.001, respectively) (see Figure 1). However, no significant relationship was found in either dependent variable (BPN satisfaction: t = 0.019, *p* = 0.985; intention to be physically active: t = 0.955, *p* = 0.341), when there were high perceived autonomy support values from the PE teacher and the father (see Figure 1). 

Considering the three-way interaction terms, only the interaction between the PE teacher, father, and peers was a significant predictor of both BPN satisfaction and autonomous motivation for LTPA in the final model (step 4, see Table 2). The three-way interaction plots mainly revealed that the lowest values of BPN satisfaction and autonomous motivation for LTPA were associated with low perceived autonomy support from the PE teacher, father, and peers, whereas, on the contrary, the highest values in those variables were associated with high autonomy support from all three sources (see Figure 1). According to the slope difference test, no significant differences were found between the magnitude of each pair of regression slopes, with the exception of the relationship between slopes 2 and 4 on BPN satisfaction in LTPA (see Table 3). The addition of the four-way interaction terms in step 4 only significantly increased the explained variance in autonomous motivation for LTPA (see Table 2).

#### 3.2.2. Hypothesis 2b: Person-Centered Approach

**Figure 2:**
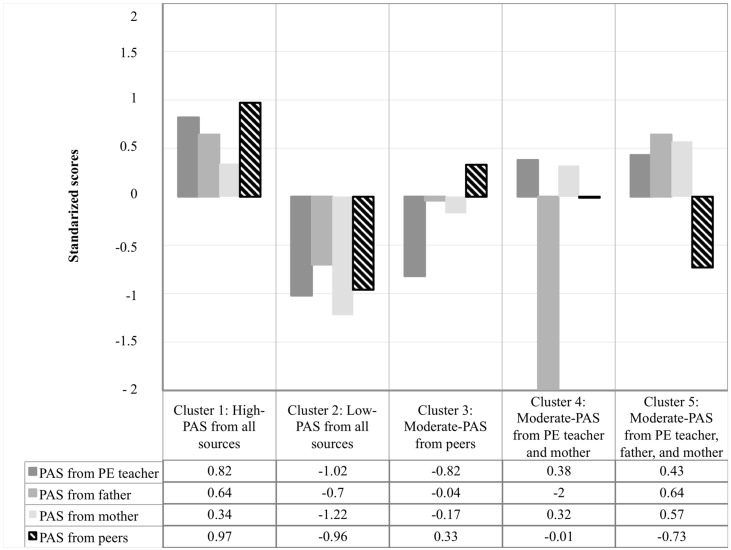
Standardized scores for autonomy support from PE teachers, father, mother, and peers for the five-cluster solution.

Regarding cluster analysis, five clusters were obtained, explaining 63%, 64%, 50%, and 59% of explained variance of autonomy support for LTPA from the PE teacher, father, mother, and peers, respectively. Other three-, four-, and six- cluster solutions explained less than 50% in some of the sources of autonomy support for LTPA [53], so they were not considered as possible solutions. The double-cross validation procedure showed good stability and replicability for the five-cluster solutions (*K* = 0.82). The five-cluster solution based on z-scores (y-axis) is presented in Figure 2. It is important to note that profiles were expressed in relative terms, rather than absolute terms, and mean scores in all study variables were above the midpoint of their respective measurement scales. Therefore, labels and characteristics used to describe profiles do not always correspond to absolute terms (See Figure 2). As observed in Table 4, although autonomy support values showed statistically significant differences between most of the five clusters, two opposite autonomy support configurations were identified, in particular, between clusters 1 and 2. Cluster 1 (*n* = 50 students, 28.10%) was comprised of adolescents who perceived the PE teacher, father, mother, and peers as highly autonomy-supportive, whereas cluster 2 (*n* = 33 students, 18.50%) was characterized by adolescents’ perceptions of low autonomy support from all these sources. The remaining three groups of adolescents (i.e., clusters 3, 4 and 5) perceived one, two or three sources as moderately autonomy-supportive, respectively. Cluster 3 (*n* = 37, 20.80%) was characterized by adolescents who perceived their peers as moderately autonomy-supportive, and their PE teacher as low autonomy-supportive. Cluster 4 (*n* = 16, 9%) was comprised of adolescents who perceived moderate autonomy-support from the PE teacher and mother, and very low autonomy support from the father. Finally, cluster 5 (*n* = 42, 23.6%) included adolescents who perceived all sources as moderately autonomy-supportive with the exception of peers who showed the opposite pattern. 

Boys and girls were equally distributed across the five-cluster solution, with the exception of the “low autonomy support” profile (11 boys and 22 girls). Chi-squared test revealed no significant association between the five-cluster solution and gender (χ^2^ [4178] = 6.734, *p* > 0.05). No significant differences were found, either, between the five-cluster solution and SES (F = 0.765; *p* > 0.05). Gender and SES were consequently not considered as covariates in further analyses. MANOVA showed a significant multivariate cluster membership effect for motivational outcomes and PA levels (Wilks’ Lambda = 0.034; F (32, 613.773) = 28.779; *p* < 0.001; η^2^_p_ = 0.570). As shown in Table 4, the “high autonomy support” group (Cluster 1) reported significant higher motivational outcomes and PA levels than all other groups, with the exception of the “moderate autonomy support from the PE teacher, father, and mother” group in terms of PA levels. The “low autonomy support” group showed significant lower motivational outcomes and PA levels than the other groups, with the exception of the “moderate autonomy support from the PE teacher and mother” group in terms of PA levels. No significant differences were found in motivational outcomes and PA levels among the profiles of adolescents who perceived moderate autonomy support from one, two or three sources (Cluster 3, 4, and 5).

## 4. Discussion

Grounded in SDT, this study investigated the role of different school community agents on motivational outcomes in a LTPA context and PA levels in a sample of adolescents, and whether the additional combination of autonomy-supportive relationships displays more beneficial effects. The main findings were as follows: (a) autonomy support from the mother, father, peers, and PE teacher significantly and positively predicted BPN satisfaction and autonomous motivation for LTPA, intention to be active, and PA levels; (b) adolescents with autonomy support from all sources reported the most optimal pattern of outcomes; (c) adolescents with moderate to high support from at least one relevant source reported higher motivational outcomes in a LTPA context and PA levels than adolescents with low levels of support from different sources; (d) no differences in motivational outcomes in a LTPA context and PA levels were found among profiles with one, two or three sources of support; (e) peers were identified as the most influential source of autonomy support for LTPA. 

Consistent with the hypothesis 1 and in agreement with the tenets of SDT, autonomy support for LTPA from the PE teacher, mother, father, and peers was significantly and positively related to students’ motivational outcomes in a LTPA context and PA levels (with the exception of the mother for PA levels). These findings highlight that school-based motivational interventions to increase adolescents’ PA levels should involve at least these school community agents. Based on the magnitude of the standardized regression coefficients, peers’ autonomy support was found to be the most salient source of autonomy support in adolescents’ PA behavior, in particular, intention to be physically active and PA levels. Parents and the PE teacher have the potential to satisfy adolescents’ BPN satisfaction in LTPA, which has, in turn, been linked to more autonomous motivation [19]. Meanwhile, peers have been closely linked with PA involvement during adolescence [5], which could explain our findings. 

Our results are in line with most of the previous studies that showed that students’ perception of autonomy support from the PE teacher, and/or parents, and/or peers were independently and significantly associated in a positive way with BPN satisfaction [10], autonomous motivation for PA [16], intention to be physically active [17], and objective PA levels [19]. As far as parental figures are concerned, students’ motivational outcomes in LTPA were significantly and positively predicted by mothers’ and fathers’ autonomy support, which seems to be consistent with the only study to date [16]. However, whereas the aforementioned study showed a significantly positive association between mothers’ and fathers’ autonomy support for PA and self-reported PA levels [16], this study showed that the father was the only parental figure that significantly and positively predicted PA levels. Therefore, school-based PA interventions need to include not only mothers, as seen in most previous studies, but also fathers as key figures in PA promotion [56]. A moderate positive correlation (*r* = 0.36) was found in this study between autonomy support from mothers and fathers, which suggests, in line with other studies [16], that adolescents may differentiate between mothers’ and fathers’ roles in PA promotion. It has been suggested that mothers are more likely to provide logistic support, while fathers tend to be more involved in children’s PA participation (i.e., modeling and involvement support) [57], which could explain the differences observed with respect to PA levels. Further studies should consider evaluating not only parental autonomy support for PA, but also mothers’ and fathers’ modeling and involvement support for PA as well as their own PA levels to refute this explanation. The measure of BPN satisfaction in PA, and the use of objective PA levels expands on previous research [16] and helps to improve our understanding about the different roles that the support of mothers and fathers have on adolescents’ motivational outcomes and PA levels. 

Regarding teachers in the educational context, our results are also consistent with previous studies that showed that although PE teachers are not directly present in the PA context, they can also influence students’ motivational outcomes in a LTPA context and PA levels [16], particularly in the satisfaction of BPNs. However, contrary to our hypothesis, the univariate and multivariate regression analysis showed that autonomy support for PA from the tutor and teachers from different areas did not seem to have a significant influence on promoting motivational outcomes in a LTPA context and PA levels. One possible explanation for these results may be found in the new Spanish educational curriculum of Secondary Education (for further review: https://www.mecd.gob.es/educacion-mecd/) where the PE curriculum is only directly and explicitly related to health promotion behaviors (e.g., PA). Future changes in the curriculum content should be aimed at developing more comprehensive and holistic approaches to school health promotion, involving all areas and school community agents. Given that the use of physically active teaching methods, such as classroom-based PA programs, has revealed promising results in terms of learning and PA promotion [58], some elements of this innovative approach should be considered. 

This study also analyzed whether the synergistic interplay of the sources of autonomy support that showed a significant independent influence on students’ motivational outcomes in a LTPA context and PA levels (i.e., PE teacher, mother, father, and peers) could explain additional variance in selected outcomes. Partially consistent with our hypotheses 2a, while the inclusion of two- and three-way interactions of these four sources of autonomy support for PA increased the amount of explained variance in motivational outcomes in a LTPA context and PA levels, above and beyond the independent effects, the four-way interaction only showed an increase in the percentage of variance in autonomous motivation for LTPA. The magnitude of the interactive effects that emerged in motivational outcomes in LTPA (total ∆*R*^2^ from 0.07 in intention to be active to 0.10 in autonomous motivation for LTPA) was even greater than reported in previous studies on motivational outcomes in the youth sports domain (total ∆*R*^2^ from 0.03 to 0.07) [24,27,28] or in social science literature (∆*R*^2^ account for approximately 1% to 3% of the total variance) [59]. 

As detected from simple slope tests of two-way interactions, the predicted change in BPN satisfaction in LTPA and intention to be physically active scores when moving from low to high autonomy support from the father was positive and statistically significant at low but not at high values of autonomy support from the PE teacher. Given that the lowest values of BPN satisfaction in LTPA and intention to be physically active were found when there was low perceived autonomy support from the PE teacher and father, it is particularly important that at least one of these two sources, in particular the father, should provide autonomy support for PA. Additionally, the three-way interaction plots revealed that the lowest values of BPN satisfaction and autonomous motivation in LTPA were found when there was low perceived autonomy support from the PE teacher, father, and peers, whereas the highest values were shown when adolescents perceived high autonomy support from all three sources. However, the results must be interpreted with caution because the slope difference test only showed significant differences between the magnitude of two out of six slopes on BPN satisfaction in LTPA. These findings would be in line with the additive model, that establishes that each additional source of support matters in psychological outcomes. However, other studies on the youth sports domain have found that interactions from at least two or three social agents could compensate the lack of support from the other agents to sustain higher levels of competence [27], self-determined motivation [27], and continued sport participation [28]. The fact that the addition of four-way interaction terms only reported a slightly significant increase in autonomous motivation for LTPA demonstrates the complex relationship dynamics embedded in school-community partnerships. Given that the current study is the first to examine the interactions between the PE teacher, mother, father, and peers in motivational outcomes in a LTPA context and PA levels, further qualitative studies are needed to better understand the synergistic interplay between these school community agents for PA promotion.

To examine the potential co-occurrence of autonomy support for PA between the PE teacher, mother, father, and peers, and to analyze the differences in terms of motivational experiences in a LTPA context and PA levels across profiles, a person-centered approach was also used. Consistent with our hypotheses 2b and previous studies [25,30,31], we identified “high autonomy support” and “low autonomy support” profiles perceived by the four social agents. Likewise, several mixed autonomy profiles, characterized by moderate autonomy support from one, two or up to three social agents, were identified. Descriptive results highlight that, although a small group of adolescents perceived high or low autonomy support from all potential sources of the school community respectively, most of the adolescents reported between one and three sources of autonomy support. Differences found in the literature with respect to sample characteristics (e.g., adolescents, university students), settings (e.g., education, sport), agents involved (e.g., parents, coaches, peers), types of support from each agent (e.g., autonomy support, peer acceptance, and peer quality), and different measurements of the same variable across these social agents (e.g., autonomy support from mother and teacher with different instruments) make it difficult to compare our profiles with other studies, as well as the role played by each social agent in the outcomes of this study.

Consistent with our expectations, adolescents from the “high autonomy support” group reported the greatest motivational outcomes and PA levels. One exception was observed with adolescents from the “moderate autonomy support from PE teacher, father, and mother” group in terms of PA levels. Hence, all sources need to be autonomy-supportive to achieve higher motivational experiences in LTPA among adolescents (additive model). However, it should be noted that the lack of support from the most influential agent (i.e., peers) in this sample is compensated by other potential sources of the school community (i.e., PE teacher, father, and mother) who provided high autonomy support (threshold model). No differences in PA levels between these two mentioned profiles could be explained because fathers continue to be involved in their children’s PA participation [57]. In line with our hypotheses, adolescents from the “low autonomy support” group reported the lowest motivational outcomes and PA levels (additive model), with the exception of adolescents from the “moderate autonomy support from PE teacher and mother” group in terms of PA levels (threshold model). The weak, or lack of, association found in the present study between PE teacher and mothers with respect to PA levels could explain the lack of differences in PA levels between these two profiles. However, it is important to highlight that our purpose is not to suggest that the PE teacher and the mother are not important agents to promote PA participation. On the contrary, we would like to explicitly point out that they should be considered when designing school-based PA interventions as key sources of support. 

Finally, consistent with the threshold model, no differences were found in motivational outcomes in a LTPA context and PA levels between the groups of students who perceived moderate autonomy support from one, two or three sources of support. Although, in this study, we only found three mixed autonomy support profiles, these findings may indicate that the number of sources of autonomy support for PA is not necessarily better if those sources of support do not have enough influence on adolescents’ motivation and PA levels. Consistent with our hypothesis, values of the “moderate autonomy support from peers” group, evidence that peers can buffer the negative effects of autonomy support from the PE teacher and parents. Our findings are in line with previous studies that emphasize the key role of peers in PA promotion in early adolescence [60]. Given that only a small proportion of adolescents meet PA guidelines (i.e., 60 minutes of daily MVPA), in the Spanish context [61], our results suggest the adoption of a global approach that should involve not only the largest number of school community agents but also the most relevant social agents in adolescence.

Several strengths should be mentioned. Firstly, the use of accelerometers to assess PA levels is one of the major strengths of this study. Secondly, another strength of this study was the evaluation of autonomy support for PA from up to six significant agents with the same self-reported instrument (i.e., PASSES), also considering the assessments of mothers and fathers, separately. Thirdly, another strength of this study was the specific age of the adolescents (as similar as possible), as social support changes throughout adolescence [5]. Finally, the last strength of this study is the use of the variable- and person-centered approaches, and the examination of the full motivational sequence of SDT. Limitations and future directions are also discussed. Firstly, only self-reported questionnaires were used to capture the other psychological variables of the study. Future studies should introduce complementary measures (i.e., qualitative methodology, and teachers’, parents’ and peers’ perception of their autonomy support and their motivational outcomes for the LTPA context and PA levels) to triangulate results and strength these findings. It would also be interesting to examine autonomy support for PA from other socialization figures, such as siblings, grandparents or close friends, or to assess other types of support for PA (e.g., logistic, modeling, involvement, etc.). Secondly, the sample of this study was recruited from only two high schools of a region in Spain, which may introduce some bias in the generalization of these findings. A representative sample of adolescents with similar and different ages from different types of schools, countries, and cultures could be analyzed to refute these findings. For example, the role of the teacher or tutor to promote PA outside school could be different across countries, due to differences in educational curriculums or cultural values. Thirdly, given the cross-sectional nature of this study, it was not possible to test the direction of causality between variables. Further, due to the complex nature of possible combinations of autonomy-supportive relationships (i.e., 16 possible combinations), it is not possible to fully analyze the additive and threshold models using cluster analysis, which makes it difficult to discuss all options for both approaches. Longitudinal or experimental designs to examine the direction of the proposed associations are an important avenue for future research. 

## 5. Conclusions

This study provides insight into the role of different school community agents in PA-related outcomes and the question of whether the number of autonomy-supportive relationships matters in students’ motivational experiences in a LTPA context and PA levels. The present study reveals that receiving autonomy support for PA from at least one salient source may be better than low levels of support from all sources in terms of students’ motivational outcomes and PA levels. Although the lack of differences between one, two, and three sources of support profiles suggests that more is not always better, the adoption of a whole-of-school approach, involving the mother, father, PE teacher, and peers, seems to be the best way to empower adolescents to be active inside and outside school. The findings also provided evidence that peers were perceived as the most relevant source of support in adolescents’ motivational outcomes and PA levels, and may compensate the low perceived support from other social agents. Considering educational agents, only the PE teachers seem to have a significant influence on promoting motivational outcomes in a LTPA context and PA levels. Nevertheless, teachers and tutor should not be forgotten due to their potential role as PA promoters in a whole-school approach. These findings invite a reconsideration of the school community agents that should be involved in the design of successful school-PA interventions.

## Figures and Tables

**Table 1:** Descriptive statistics and Pearson’s bivariate correlations between study variables.

Study Variables	M	SD	1	2	3	4	5	6	7	8	9	10
1. PAS from PE teachers	4.46	1.25	—									
2. PAS from teachers	3.57	1.28	0.29 **	—								
3. PAS from father	5.34	1.53	0.31 **	0.10	—							
4. PAS from mother	5.43	1.27	0.32 **	0.03	0.36 **	—						
5. PAS from peers	3.89	1.66	0.29 **	0.12	0.17 *	0.13	—					
6. PAS from tutors	2.65	1.25	0.15 *	0.03	0.05	0.15 *	0.18 *	—				
7. BPN satisfaction in LTPA	3.67	0.67	0.42 **	0.20 **	0.36 **	0.32 **	0.41 **	0.09	—			
8. Autonomous motivation for LTPA	2.42	0.67	0.36 **	0.18 **	0.37 **	0.37 **	0.33 **	0.08	0.56 **	—		
9. Intention to be physically active	5.17	1.49	0.38 **	0.17 *	0.40 **	0.40 **	0.40 **	0.16 *	0.52 **	0.54 **	—	
10. Objective PA levels	45.45	15.60	0.35 **	0.10	0.34 **	0.28 **	0.37 **	0.10	0.28 **	0.34 **	0.46 **	—

Note: * *p* ≤ 0.05; ** *p* ≤ 0.001.

**Table 2:** Multivariate hierarchical regression analysis with autonomy support for PA from the PE teacher, father, mother, peers, and tutors, predicting need satisfaction and autonomous motivation for LTPA, intention to be physically active, and objective PA levels.

Outcome Variable Step (*df*)	F	*R*^2^	∆*R*^2^	Standarized Regression Coefficients															
				1-way					2-way						3-way				4-way
				PE	FA	M	P	T	PExFA	PExM	PExP	FAxM	FAxP	MxP	PExFAxM	PExFAxP	PExMxP	FAxMxP	PExFAxMxP
BPN satisfaction in LTPA																			
Step 1 (5177)	17.619 *	0.34		0.22 *	0.19 †	0.14 †	0.29 *	0.08											
Step 2 (11,177)	10.052 *	0.40	0.06	0.20 *	0.18 *	0.10	0.31 *	0.08	−0.21	0.07	−0.01	−0.11	0.06	−0.00					
Step 3 (15,177)	8.063 *	0.43	0.03	0.15 †	0.13	0.12	0.32 *	0.06	−0.24 *	0.09	−0.04	−0.04	0.08	−0.00	0.08	0.17 *	0.04	−0.17	
Step 4 (16,177)	7.649 *	0.43	0.00	0.15 †	0.15	0.10	0.32 *	0.06	−0.24 *	0.06	−0.04	−0.03	0.04	−0.03	0.06	0.19 †	0.00	−0.12	0.11
Autonomous motivation for LTPA																			
Step 1 (5177)	14.812 *	0.30		0.14 †	0.20 *	0.23 *	0.22 *	0.08											
Step 2 (11,177)	8.487 *	0.36	0.06	0.13	0.20 *	0.18 †	0.23 *	0.07	−0.07	−0.04	−0.02	−0.12	0.17 †	−0.08					
Step 3 (15,177)	6.731 *	0.38	0.02	0.10	0.17 †	0.22 †	0.21 *	0.05	−0.07	−0.06	−0.03	−0.09	0.15 †	−0.04	−0.02	0.20 †	0.00	−0.09	
Step 4 (16,177)	6.688 *	0.40	0.02	0.10	0.20 †	0.18 †	0.21 *	0.06	−0.08	−0.10	−0.03	−0.08	0.08	−0.08	−0.05	0.22 †	−0.05	0.01	0.20 *
Intention to be physically active																			
Step 1 (5177)	19.713 *	0.36		0.14 †	0.22 *	0.24 *	0.29 *	0.06											
Step 2 (11,177)	10.305 *	0.41	0.05	0.16 †	0.20 *	0.21 *	0.28 *	0.04	−0.20 *	−0.06	0.03	0.08	0.11	−0.07					
Step 3 (15,177)	7.813 *	0.42	0.02	0.16 †	0.26 *	0.26 *	0.28 *	0.04	−0.20 *	−0.03	0.00	−0.07	0.09	−0.00	−0.21	0.00	0.06	−0.09	
Step 4 (16,177)	7.282 *	0.42	0.00	0.16 †	0.26 *	0.26 *	0.28 *	0.04	−0.20 *	−0.04	0.00	−0.07	0.08	−0.00	−0.21	0.00	0.05	−0.08	0.01
Objective PA levels																			
Step 1 (5177)	12.498 *	0.27		0.17 †	0.20 *	0.12	0.27 *	−0.00											
Step 2 (11,177)	5.846 *	0.28	0.01	0.16	0.21 *	0.11	0.29 *	−0.00	−0.08	0.08	−0.05	−0.05	0.00	−0.04					
Step 3 (15,177)	4.288 *	0.29	0.01	0.16	0.21 †	0.16	0.31 *	−0.00	−0.09	0.10	−0.05	−0.07	−0.00	−0.03	−0.05	0.01	−0.04	−0.05	
Step 4 (16,177)	4.087 *	0.29	0.00	0.16	0.23 *	0.13	0.31 *	0.00	−0.10	0.08	−0.05	−0.06	−0.05	−0.06	−0.07	0.02	−0.07	0.00	0.11

Note: *df* = Degrees of freedom; PE = Physical education; FA = Father; M = Mother; P = Peers; T = Teachers from different areas; † *p* ≤ 0.05; * *p* ≤ 0.001.

**Table 3:** Simple slope comparisons for the three-way interactions (autonomy support from PE teachers, father, and peers) of BPN satisfaction in LTPA and autonomous motivation for LTPA.

Slope Difference	BPN Satisfaction in PA		Autonomous Motivation for PA	
	t *	*p* *	t *	*p* *
1 and 2	0.268	0.789	0.182	0.856
1 and 3	−0.638	0.524	−0.090	0.928
1 and 4	−0.520	0.604	−0.355	0.723
2 and 3	−1.732	0.085	−0.502	0.616
2 and 4	−2.075	0.040	−1.420	0.158
3 and 4	−0.366	0.715	−0.580	0.562

Note: Number listed in slope comparisons corresponds to the number listed in Figure 1; t * = t-value for slope difference; *p* * = *p*-value for slope difference.

**Table 4:** Mean differences in motivational outcomes in a LTPA context and PA levels according to cluster membership.

Variables	Cluster 1: High-PAS from All Sources	Cluster 2: Low-PAS from All Sources	Cluster 3: Moderate-PAS from Peers	Cluster 4: Moderate-PAS from PE Teacher and Mother	Cluster 5: Moderate-PAS from PE Teacher, Father, and Mother	*F*-Value	(η^2^_p_)
PAS from PE teacher							
Raw scores	5.57 (0.12) ^a^	3.07 (0.15) ^b^	3.34 (0.14) ^b^	4.97 (0.21) ^ac^	5.04 (0.13) ^c^	62.187 ***	0.59
Z-scores	0.82 (0.09) ^a^	−1.02 (0.11) ^b^	−0.82 (0.10) ^b^	0.38 (0.16) ^ac^	0.43 (0.10) ^c^		
PAS from father							
Raw scores	6.32 (0.12) ^a^	4.22 (0.15) ^b^	5.25 (0.14) ^d^	2.20 (0.21) ^c^	6.31 (0.13) ^a^	96.262 ***	0.69
Z-scores	0.64 (0.07) ^a^	−0.70 (0.09) ^b^	−0.04 (0.09) ^d^	−2.00 (0.13) ^c^	0.64 (0.08) ^a^		
PAS from mother							
Raw scores	5.88 (0.14) ^a^	3.87 (0.17) ^b^	5.22 (0.16) ^c^	5.84 (0.24) ^a^	6.16 (0.15) ^a^	52.519 ***	0.50
Z-scores	0.34 (0.11) ^a^	−1.22 (0.13) ^b^	−0.17 (0.12) ^c^	0.32 (0.19) ^a^	0.57 (0.12) ^a^		
PAS from peers							
Raw scores	5.54 (0.15) ^a^	2.30 (0.18) ^b^	4.47 (0.17) ^c^	3.89 (0.26) ^c^	2.69 (0.16) ^b^	64.571 ***	0.59
Z-scores	0.97 (0.09) ^a^	−0.96 (0.11) ^b^	0.33 (0.10) ^c^	−0.01 (0.15) ^c^	−0.73 (0.09) ^b^		
BPN satisfaction in LTPA	4.17 (0.08) ^a^	3.07 (0.10) ^b^	3.60 (0.09) ^c^	3.67 (0.14) ^c^	3.63 (0.08) ^c^	18.332 ***	0.29
Autonomous motivation for LTPA	2.90 (0.07) ^a^	1.76 (0.09) ^b^	2.29 (0.09) ^c^	2.34 (0.13) ^c^	2.49 (0.08) ^c^	22.116 ***	0.33
Intention to be physically active	6.22 (0.17) ^a^	3.78 (0.21) ^b^	4.91 (0.20) ^c^	4.93 (0.31) ^c^	5.35 (0.19) ^c^	19.410 ***	0.31
PA levels	53.79 (1.97) ^a^	32.05 (2.42) ^b^	44.40 (2.29) ^c^	42.43 (3.48) ^bc^	47.78 (2.15) ^ac^	12.100 ***	0.21

Note: PAS = Perceived autonomy support. Standard errors are reported in parenthesis; A group mean is significantly different from another mean if they have different superscripts; *** = *p* < 0.001.
